# The shifting paradigm to social media in neurosurgery

**DOI:** 10.1186/s41016-025-00416-4

**Published:** 2025-10-31

**Authors:** Ismail Bozkurt, Bipin Chaurasia

**Affiliations:** 1 Department of Neurosurgery, Medical Park Ankara Hospital, Ankara, Turkey; 2 Department of Neurosurgery, Faculty of Medicine, Yuksek Ihtisas University, Ankara, Turkey; 3 Department of Neurosurgery, Neurosurgery Clinic, Birgunj, 44300 Nepal; 4 Department of Neurosurgery, College of Medical Sciences, Bharatpur, 44300 Nepal

## Background

This article examines the evolving role of social media in Neurosurgery. It focuses on the transformative role that digital media have taken in altering how neurosurgical information is communicated, collaborations are formed, and professional selves are constructed. Social media is no longer peripheral but an integral part of modern academic life [1].

With the extensive reach of social media, we would like to highlight an essential aspect deserving special mention—the functionality of *Neurosurgery Cocktail*, currently the largest neurosurgical social media group in Facebook (31 K Members), Instagram (3902 followers), X (29 K followers), Telegram (10 K members), LinkedIn (2 K), Threads, WeChat, and WhatsApp (15 K) [2, 3]. Since its inception (2016), *Neurosurgery Cocktail* has evolved from a mere discussion group to an unparalleled forum for case consultation, mentorship, education, and collaborative research. The resilience of such sites has been especially evident during the era of COVID, as restrictions on travel and in-person conferencing mandated a hasty broadening of remote involvement [4, 5]. In parallel, other platforms have played a complementary role. Zoom, Microsoft Teams, and similar webinar-based applications became essential for structured academic meetings, virtual conferences, and mentorship during COVID-19. In addition, professional societies such as AANS, WFNS, and AO Spine organized regular online educational sessions, while YouTube channels, podcasts, and journal clubs on X provided additional opportunities for asynchronous learning and discourse. Together, these diverse digital platforms reflect a broader shift in neurosurgical education and collaboration beyond a single community.

*Neurosurgery Cocktail* and other sites served as a lifeline amid the pandemic for neurosurgeons everywhere, delivering instantaneous consultation on complex cases and facilitating elder mentorship that otherwise may have been irretrievable. The ability to accumulate diverse opinions on surgical techniques, exotic pathologies, and treatment dilemmas with immediate worldwide access has potentially improved patient care by enabling rapid consultation and diverse perspectives. More importantly this virtual space has fueled a new generation of scholarship collaboration. On social media, communities of far-flung neurosurgeons have congregated to produce high-impact papers, a phenomenon amplified by the digital age [6, 7].

While prior publications have explored the general influence of social media on neurosurgery, this report uniquely focuses on the sustained evolution and academic contributions of *Neurosurgery Cocktail*—an in-depth case study of the most active and far-reaching neurosurgical social media community to date.

## Contributions of *Neurosurgery Cocktail* to academic neurosurgery

Several publications have been a direct result of the collaborations facilitated by *Neurosurgery Cocktail* [4]. For instance, an influence study on the platform indicated that as of December 2022, the community comprised 29,524 members from over 100 nations with 12.7 daily posts over 60 days. The content ranged from discussing clinical cases to knowledge sharing, reflecting the potential of the platform in enabling global neurosurgical communication and education [3]. Another survey of 1119 neurosurgeons from 104 countries showed that most respondents believed social media was beneficial for neurosurgery [8]. The survey discovered that platforms like *Neurosurgery Cocktail* have possibilities of networking, acquiring information about new studies or conferences, and quick knowledge transfer, thus facilitating professional growth and collaboration.

*Neurosurgery Cocktail* operates as an open-access platform where neurosurgeons frequently post de-identified radiological images or intraoperative videos to seek real-time input from peers across the globe. For instance, a complex skull base tumor case shared by a neurosurgeon in India prompted expert advice from specialists in Canada and Japan within hours, altering the surgical approach and improving outcomes. The platform also fosters intergenerational mentorship; retired professors often provide seasoned perspectives, guiding junior surgeons on nuanced management strategies that may not be found in textbooks. These discussions, archived and searchable, serve as living educational repositories and often influence real-time decision-making, potentially contributing to optimized and individualized patient care strategies.

Between January 2020 and December 2023, *Neurosurgery Cocktail* recorded approximately 1200 case-specific discussion threads involving radiological images, operative videos, and diagnostic dilemmas. These posts consistently elicited prompt responses, with a median of 14.6 expert-level comments per case (range, 3–52), and an average response latency of 2.8 h. Notably, over 65% of the engagements involved board-certified neurosurgeons or academic faculty, suggesting a high level of peer-to-peer discourse quality. Data were extracted from platform analytics and verified through manual review of posts from January 2020 to August 2025. Posts originating from resource-limited settings, such as sub-Saharan Africa or Southeast Asia, showed significant engagement, suggesting a role in addressing regional disparities in subspecialty access. Additionally, 23 published articles in PubMed-indexed journals (as of early 2024) list contributors who initially connected through case discussions on *Neurosurgery Cocktail*, indicating a measurable academic yield from informal online collaboration. This data was identified through contributor acknowledgments and platform records and highlights the platform’s value not only as an educational resource but as a digital ecosystem fostering real-time consultation, equitable access, and collaborative scholarship. Comparable benefits have been reported in other settings; for instance, society-led webinars and virtual journal clubs have also demonstrated their potential to foster mentorship, rapid information exchange, and scholarly collaborations.

## Social media knowledge versus evidence-based practice

The immediacy of social media platforms enables neurosurgeons to access diverse perspectives on complex cases within hours, a feature that is particularly valuable in urgent clinical contexts. However, this rapid exchange of opinions is not equivalent to evidence-based medicine. Unlike peer-reviewed literature, information shared on social media lacks formal validation and may occasionally conflict with established guidelines. Applying such advice directly to patient care without adequate scrutiny carries potential medicolegal ramifications, particularly in cases involving high-stakes decision-making.

Social media discussions should therefore be considered a valuable adjunct to, rather than a substitute for, traditional evidence-based practice. While they can illuminate gray zones of clinical management, refine decision-making, and foster critical dialogue, they cannot replace the structured rigor of clinical trials, published guidelines, and institutional protocols. Moreover, knowledge acquisition through digital interaction must be balanced with the development of surgical dexterity. Operative proficiency requires cadaveric dissections, simulation-based training, and supervised mentorship in the operating room, skills that cannot be transmitted through online dialogue alone. Thus, social media occupies an important complementary role, enhancing theoretical understanding and collaborative learning while underscoring the necessity of hands-on surgical education.

## Limitations and challenges of social media platforms

An additional challenge lies in objectively measuring the educational and professional impact of social media engagement. Comparative studies, evaluating neurosurgeons who actively use such platforms versus those who do not are lacking. Rigorous multicenter investigations are needed to define the tangible domains in which social media contributes to skill development, decision-making, or career advancement. Despite advantages, inherent limitations and drawbacks associated with the use of social media websites in neurosurgery exist (Table 1).

**Table 1:** Limitations and challenges of social media platforms

• Privacy concerns: A significant issue identified in the analysis of *Neurosurgery Cocktail* was the potential for privacy violations. In a quality assessment of 173 clinical cases presented on the platform, issues with privacy were recorded in 50.9% of cases, highlighting the need for stringent measures to protect patient confidentiality. (https://pubmed.ncbi.nlm.nih.gov/37383446/) [3]
• Quality of shared content: The same study found that imaging was considered insufficient in 39.3% of cases, clinical data were inadequate in 53.8%, and follow-up data were missing in 60.7%. These findings suggest that while social media facilitates rapid information exchange, the quality and completeness of shared medical information can be suboptimal. (https://pubmed.ncbi.nlm.nih.gov/37383446/) [3]
• Misinformation: The rapid dissemination of information on social media platforms can lead to the spread of misinformation, which may adversely affect clinical decision-making and patient outcomes
• Professional boundaries: Maintaining professionalism on social media is challenging, as the lines between personal and professional content can blur, potentially impacting the credibility of healthcare professionals

In conclusion, while platforms like *Neurosurgery Cocktail* have significantly promoted global neurosurgical collaboration, education, and research, it is essential to address the challenges they pose. Modifying guidelines for content quality, maintaining patient confidentiality, and promoting digital professionalism are fundamental steps toward maximizing social media advantages in neurosurgery [9].

As neurosurgery becomes increasingly integrated with digital media, caution must be exercised to mitigate risks such as misinformation, confidentiality breaches, and commercialization. When used responsibly, platforms such as *Neurosurgery Cocktail*, along with webinars, YouTube channels, and society-based forums, demonstrate the significant potential to enhance education, patient management, and research collaboration when used responsibly. Future studies should aim to quantify these contributions and guide the development of professional standards for responsible use.

## Data Availability

No datasets were generated or analysed during the current study.
